# The Important Molecular Markers on Chromosome 17 and Their Clinical Impact in Breast Cancer

**DOI:** 10.3390/ijms12095672

**Published:** 2011-09-05

**Authors:** Wei Zhang, Yingyan Yu

**Affiliations:** 1 Department of Surgery, School of Medicine, The Ninth People’s Hospital of Shanghai Jiao Tong University, Shanghai 200011, China; 2 Department of Surgery, School of Medicine, Shanghai Ruijin Hospital of Shanghai Jiao Tong University, Shanghai 200025, China; E-Mail: yingyan3y@yahoo.com.cn

**Keywords:** chromosome 17, biomarkers, breast cancer

## Abstract

Abnormalities of chromosome 17 are important molecular genetic events in human breast cancers. Several famous oncogenes (HER2, TOP2A and TAU), tumor suppressor genes (p53, BRCA1 and HIC-1) or DNA double-strand break repair gene (RDM1) are located on chromosome 17. We searched the literature on HER2, TOP2A, TAU, RDM1, p53, BRCA1 and HIC-1 on the Pubmed database. The association of genes with chromosome 17, biological functions and potential significance are reviewed. In breast cancer, the polysomy 17 (three or more) is the predominant numerical aberration. HER2 amplification is widely utilized as molecular markers for trastuzumab target treatment. Amplified TOP2A, TAU and RDM1 genes are related to a significant response to anthracycline-based chemotherapy, taxane or cisplatin, respectively. In contrast, p53, BRCA1 and HIC-1 are important tumor suppressor genes related to breast carcinogenesis. This review focused on several crucial molecular markers residing on chromosome 17. The authors consider the somatic aberrations of chromosome 17 and associated genes in breast cancer.

## 1. Introduction

**Figure 1:**
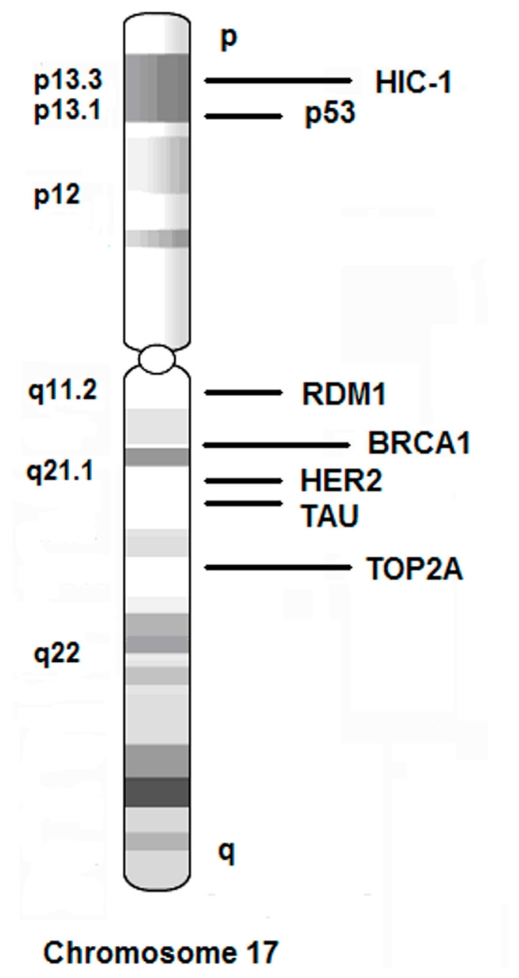
Schematic diagram of chromosome 17 and several important molecular markers for breast cancer. Gene names are listed on the right side and resided regions are listed on the left side.

In chromosome 17, several important genes are associated with breast cancer, including oncogenic genes HER2, TOP2A and TAU, tumor suppressive genes p53, BRCA1, and HIC-1, as well as DNA double-strand break repair and recombination gene RDM1 (Figure 1). In addition, abnormalities of chromosome 17 are important molecular genetic events in tumorigenesis, especially in breast cancer [1]. Both monosomy or polysomy (three or more) of chromosome 17 can be observed in breast cancer. Recently, a growing list of studies suggested that several genes residing on chromosome 17 are linked to cancer initiation, progression and therapeutic response. A better understanding of the chromosome 17 abnormalities will help to select patients who may respond well to some therapies. The examination of HER2 expression and copy number amplification (17q21.1) is widely utilized for predicting therapeutic response for trastuzumab (Herceptin) not only in breast cancer, but also in malignancies of the gastrointestinal tract [2–5].

## 2. HER2

The HER2 gene is located on 17q21.1, which encodes the human epidermal growth factor receptor 2 oncogene, a transmembrane tyrosine kinase receptor. Currently, a series of HER2-targeting agents are developed, including trastuzumab, pertuzumab, ertumaxomab and lapatinib. HER2 gene amplification or protein overexpression is encountered in approximately 25% of newly diagnosed breast cancers. Although the incidence and significance of HER2 amplification in lobular carcinoma are lower than that in ductal carcinoma, the HER2 amplification is still a significant adverse prognostic factor in lobular breast cancer [6]. One report proposed that tumor-initiating cells (side population fraction by cytometry, SP) of breast cancer disclosed significant HER2 expression. The SP fraction was decreased by HER2 inhibitors, strengthening the contribution of targeting HER2 to SP phenotype. The finding indicates an important role of HER2 in regulating the tumor-initiating cells in breast cancer [7]. A meta-analysis for cohort randomized trials on women with HER2-positive early breast cancer disclosed that trastuzumab-based adjuvant chemotherapy derived benefit in disease-free survival, overall survival and recurrence to adjuvant chemotherapy, but did worse in recurrence on the central nervous system compared to the controls [8].

Fluorescence *in situ* hybridization (FISH) is often used for detecting chromosome 17 or HER2 gene aberrations. Dual-color FISH is a method for combining analysis of chromosome ploidy and gene copy numbers by centromeric 17 and target gene probes. PathVision dual-color FISH Kit is often used for detecting chromosome 17 and copy number of HER2 target gene simultaneously, whereas, HercepTest Kit is used for detecting HER2 protein (ERBB2) immunohistochemically. Amplification of the HER2 gene is present in about 25% of breast cancer and leads to an overexpression of the protein that made it possible to develop a targeted therapy by the monoclonal antibody trastuzumab. However, the suitability of target therapy depends on the stringent assessment of the gene status in tumors. Only patients whose tumor shows a high expression of the HER2 protein or an amplification of the gene are eligible for target therapy. The candidate gene is considered as amplification when showing more than six HER2 copies per nucleus, or with a ratio of HER2 to centromere 17 greater than 2.2. Clusters of signals are characteristic of the HER2 amplifications [9].

The HER2 amplification and over-expression have been related to negative responses to conventional chemotherapy and poor prognosis but better overall survival rate for trastuzumab in breast cancer [10,11]. The American Society of Clinical Oncology and the College of American Pathologists (ASCO/CAP) proposed that HER2 negative means that a tumor with a staining score of 0 or 1+, or with a ratio of HER2 gene to chromosome 17 less than 1.8, or with four or less HER2 copy numbers per cell. The HER2 positivity is defined as a tumor with an immunostaining score of 3+ (uniform, intense membrane staining of >30% of invasive tumor cells), or with a ratio of HER2 gene to chromosome 17 greater than 2.2, or with six or greater HER2 copy numbers per cell. A tumor with an immunostaining score of 2+ should be further tested by FISH. HER2 borderline means that tumor samples with a ratio of HER2 gene to chromosome 17 of 1.8 to 2.2, or with greater than four to less than six HER2 copy numbers per cell [9,12,13]. Because HER2 and TOP2A are harbored nearby on chromosome 17q21-q22, a high concordance of the HER2 and TOP2A gene co-amplification was indicated in breast cancer [14]. The FISH method or chromogenic in situ hybridization (CISH) method can be taken for HER2 amplification detection. In breast cancer, aneusomy of chromosome 17, either monosomy (single copy per cell) or polysomy (≥3 copies per cell) is frequently observed by FISH. However, the biological significance of aneusomy of chromosome 17 remained controversial [15–19].

## 3. TOP2A

DNA topoisomerase II alpha (TOP2A) is a novel marker of cell-cycle turnover. Both of TOP2A and HER2 gene are located on chromosome 17q21-q22. TOP2A gene is more than 700 kb telomeric to HER2 and plays a key role in DNA replication. TOP2A is a major target of anthracycline activity [20]. In breast cancer, TOP2A expression has been linked to cell proliferation and HER2 protein overexpression. O’Malley *et al*. found that in patients whose tumors showed TOP2A alterations (either amplifications or deletions), treatment with cyclophosphamide, epirubicin, and 5-fluorouracil (CEF) was statistically significantly superior to treatment with cyclophosphamide, methotrexate, and 5-fluorouracil (CMF) in terms of recurrence-free survival and overall survival. The TOP2A gene alterations revealed an increase in responsiveness to anthracycline-containing chemotherapy regimens relative to non-anthracycline regimens [21]. Since the gene locus of TOP2A is closely near to HER2 gene, the amplification of TOP2A is a frequently accompanied by HER2 gene amplification. TOP2A amplification in the absence of HER2 amplification may be associated with lower histological grade and ER positivity. Orlando *et al*. assessed the 23 patients with T2-T4 ER absent and HER2 overexpression breast cancers treated with anthracycline-based chemotherapy. TOP2A was amplified in five (22%) of the tumors. In all patients with TOP2A amplification, HER2 gene amplification was also detected. The pathological complete remission was reported in three of five amplified tumors (60%), compared to that in two of 13 tumors without TOP2A amplification (15%). They proposed that in endocrine unresponsive/HER2 overexpression cases, TOP2A amplification or the polysomy of chromosome 17 are related to a significantly high remission after anthracycline-based chemotherapy [22]. TOP2A is taken as a molecular target of anthracycline drug and is potentially useful as a predictive marker of response to anthracycline therapy [23].

## 4. TAU

TAU is a microtubule-associated protein (MAPs) and located on chromosome 17q21.1. Tubulin-targeting agents affect the microtubule function to disrupt cell shape, microvesicle transportation and spindle formation. The agents include taxanes, paclitaxel, docetaxel and epothilones, which interfere with the spindle microtubule dynamics and cause cell cycle arrest. These agents are widely used in the treatment of breast cancer with benefits of overall survival and disease-free survival. Detection of TAU expression may help doctors to identify the patients who likely to benefit from taxane treatment. The low expression of TAU correlates with sensitivity to paclitaxel *in vitro*. TAU promotes microtubule assembly and stabilizes microtubules, and it is possible that TAU competes with taxanes for microtubule binding [24,25]. Researches demonstrated that high TAU mRNA expression in ER-positive breast cancer revealed an endocrine-sensitive but chemotherapy-resistant. In contrast, low TAU expression in ER-positive cancers revealed a poor prognosis with tamoxifen alone, but may benefit from taxane-containing chemotherapy [26]. In addition, Tanaka *et al*. studied the correlation of TAU expression and efficacy of paclitaxel treatment in metastatic breast cancer. They found that 57% of breast cancer tissues disclosed TAU expression. A significant difference was identified between paclitaxel effectiveness and TAU expression. Nine (60%) of 15 cases with TAU-negative expression showed favorable response to paclitaxel administration compared with three (15%) of 20 cases with TAU-positive expression [27].

## 5. RDM1

The RDM1 (RAD52 Motif 1) gene locates on 17q11.2, which encodes a protein RAD52 involved in DNA double-strand break repair and recombination event. As we know, chemical mutagens and ultraviolet lesions can lead to mutational loss of factors important for cell growth and viability. These DNA damages need to be repaired. A numbers of genes are involved into DNA repair, including excision repair, postreplication repair and recombinational/double-strand break repair. The repair of double-strand break is mediated extensively by RDA52-dependent recombination [28]. Hamimes *et al*. found that disruption of RDM1 in the chicken B cell line DT40 led to a more than threefold increase in sensitivity to cisplatin. They also found that human RDM1 transcripts are abundant in testis, suggesting a possible role during spermatogenesis and maintaining stemness of cells [29,30]. Messaoudi *et al*. studied the subcellular distribution of human RDM1 protein isoforms and proposed that RDM1 null chicken DT40 cells displayed an increased sensitivity to heat shock, compared to wild-type cells, suggesting a function for RDM1 in the heat-shock response [31]. The deficiency of DNA repair and recombination mechanisms led to a hypersensitivity to cisplatin, mitomycin C and bleomycin [32,33]. Because either bleomycin (directly) or cisplatin (DNA adduct formation) involves in the DNA double-strands break, probing the RDM1 gene aberration may provide a valuable information for action of these anticancer agents [34]. However, all the findings come from the experiments *in vitro*; the clinical significance of RDM1 assay needs to be clarified in future.

## 6. BRCA1

The breast cancer susceptibility gene BRCA1 is located on chromosome 17q12-21. BRCA1 encodes a tumor suppressor protein that acts as a negative regulator of tumor growth and is involved in DNA-damage repair. Germline mutation of BRCA1 increases the risk of developing breast cancer. Genetic factors contribute to about 5% of all breast cancer cases. In addition, the effect of BRCA1 mutation moderately increased the risk of ovarian cancer [35]. Rhiem *et al*. analyzed 105 sporadic breast carcinomas and indicated that high frequencies of loss of heterozygosity (LOH) of BRCA1 is more observed in estrogen receptor-negative carcinomas than estrogen receptor-positive carcinomas (39%:12%; p = 0.003) [36]. Tassone *et al*. constructed BRCA1-defective or BRCA1-reconstituted human breast cancer xenografts models and found that CDDP induced almost complete growth inhibition of BRCA1-defective xenograft tumor growth, while BRCA1-reconstituted xenograft tumor were only partially inhibited. They proposed that BRCA1-defective xenograft tumor express a high sensitivity to platinum-derived chemotherapy [37]. Liu *et al*. successively induced mammary tumors with features of human BRCA1-mutated basal-like breast cancer with somatic loss of BRCA1 and p53 in mice. Somatic loss of both BRCA1 and p53 resulted in the rapid and efficient formation of highly proliferative, poorly differentiated, estrogen receptor-negative mammary carcinomas, reminiscent of human basal-like breast cancer [38]. However, somatic BRCA1 mutations are rarely found in sporadic breast tumours. BRCA1 methylation has been shown to occur in sporadic breast tumours and to be associated with reduced gene expression. Thus, epigenetic silencing and deletion of the BRCA1 gene might serve as Knudson's two 'hits' in sporadic breast tumorigenesis [39–41].

## 7. P53

The tumor suppressor gene p53 is located on 17p13.1. Wild-type p53 is a checkpoint gene critical to control cell growth and to maintain genomic stability. P53 tumor suppressor plays a pivotal role in the coordination of the repair process or in the induction of apoptosis. P53 somatic alteration is described in approximately 50% of human cancers [42]. Activation of p53 may have potential value as cancer therapeutic strategy. Wild-type p53 gene encodes a phosphoprotein, which barely detectable in the nucleus of normal cells (with a short half-life of 20 min). Upon cellular stress, particularly that induced by DNA damage, p53 can arrest cell cycle progression, thus allowing the DNA to be repaired; or leading to apoptosis. In cancer cells bearing a mutant p53, this protein is no longer able to control cell proliferation, resulting in inefficient DNA repair and genetic instability. The point missense mutation of p53 is the most common change in human cancers, including colon cancer, stomach cancer, breast cancer and esophagus cancer. The mutant p53 protein is detectable protein accumulated in the nucleus of neoplastic cells (with a longer half-life of several hours). Therefore, positive p53 immunostaining represents the abnormal p53 gene. During the past few years, the dramatic progress in the molecular biology of p53 has raised the exciting prospect for cancer management. MDM2 (also named Mdm2 p53 binding protein homolog) can inactivate the p53 via binding to the transactivation domain of p53. Overexpression of this gene can result in excessive inactivation of p53 tumor suppressor protein. MDM2 has E3 ubiquitin ligase activity, which target p53 for proteosomal degradation. Modulting p53 activity with MDM2 inhibitor is a promising approach for treating cancer [43]. A small-molecule MDM2 antagonist, nutlin-3 has been developed. Cancer cells with MDM2 gene amplification are most sensitive to nutlin-3 *in vitro* and *in vivo*. The patients with wild-type p53 tumors may benefit from antagonists of the p53-MDM2 interaction [44].

## 8. HIC-1

Hypermethylated in cancer 1 (HIC-1, also named ZBTB29 or ZNF901) is a candidate tumor suppressor gene which is located at 17p13.3, a more distal region of p53 on chromosome 17p, which frequently undergoes allelic loss in breast and other human cancers. The human HIC1 gene is composed of two exons [45]. HIC-1 is ubiquitously expressed in normal tissues, but low-expressed in different tumor cells, including hepatocellular carcinoma, colorectal carcinoma, breast cancer and gastric cancer [46–54]. In breast cancer, the loss of heterozygosity at 17p13 combined with p53 gene mutation was related to survivin overexpression, a member of the inhibitor-of-apoptosis (IAP) family [55]. The DNA hypermethylation of HIC-1 was found in several kinds of tumor [56]. The HIC-1 protein expression has been linked to better outcomes in breast cancers. Although the molecular mechanism underlying HIC1-mediated transcriptional and growth suppression is currently unclear, some researchers proposed that HIC-1 targets E2F-responsive genes responsible for transcriptional regulation and growth suppression in cancer [57]. Recently, Zhang *et al*. found that HIC-1 can repress the ephrin-A1 transcription, which implicated in the pathogenesis of epithelial cancers. Restoration of HIC-1 in breast cancer cells led to a growth arrest *in vivo* [58]. One report demonstrated that HIC-1 regulates breast cancer cell responses to endocrine therapies [59]. The expression of HIC-1 is linked to p53 status. For instance, MCF-7, a breast cancer cell line with wild type p53 expressed HIC-1, but the MDAMB231 breast cancer cell line with mutant p53 did not express HIC-1, suggesting synergistic events of loss of HIC-1 expression and p53 mutation in breast cancer. The HIC-1 expression can be restored by demethylating drug 5-aza-2′-deoxycytidine in MDAMB231 cells. Therefore, restoration of HIC-1 function by demethylation may offer a therapeutic avenue in breast cancer [60].

## 9. Conclusion

We summarized the important molecular markers resided on chromosome 17 and their biological significance (Table 1). We also introduced the examining and evaluating methods for chromosome 17 and associated molecular markers. This review strengthened the clinical significance of monitoring the aberration of molecular markers on chromosome 17 in breast cancer.

## Figures and Tables

**Table 1:** Molecular markers and their functions on chromosome 17.

Gene ID	Name	Location	Functions
2064	ERBB2/HER2	17q21.1	Epidermal growth factor (EGF) receptor family of receptor tyrosine kinases. Amplification and/or overexpression have been reported in numerous cancers.
7153	TOP2A	17q21-q22	DNA topoisomerase, controls and alters the topologic states of DNA during transcription. It is associated with the development of drug resistance.
201299	RDM1	17q11.2	RAD52 protein encoded by RDM1 is involved in DNA double-strand break repair and recombination event. Disruption of the RDM1 gene resulted in an increased sensitivity to the anti-cancer drug cisplatin.
7157	P53	17p13.1	P53 responds to diverse cellular stresses to regulate target genes that induce cell cycle arrest, apoptosis, senescence, DNA repair. It is accumulated in a variety of transformed cells.
672	BRCA1	17q21	BRCA1 plays a role in maintaining genomic stability. It acts as a tumor suppressor. BRCA1 combines with other tumor suppressors, to form a BRCA1-associated genome surveillance complex (BASC). Mutations in this gene are responsible for approximately 40% of inherited breast cancers and more than 80% of inherited breast and ovarian cancers.
3090	HIC-1	17p13.3	Hypermethylated in cancer 1, a candidate tumor suppressor gene which undergoes allelic loss in breast and other human cancers. The human HIC-1 gene is a target gene of p53.
4137	TAU	17q21.1	Microtubule-associated protein TAU (MAPT), functions to keep cell shape, microvesicle transportation and spindle formation. Interfering spindle microtubule dynamics will cause cell cycle arrest and apoptosis. TAU detection helps to identify those patients who are most likely to benefit from taxane treatment and resistant to paclitaxel treatment.
